# An Edge AI Approach for Low-Power, Real-Time Atrial Fibrillation Detection on Wearable Devices Based on Heartbeat Intervals

**DOI:** 10.3390/s25237244

**Published:** 2025-11-27

**Authors:** Eliana Cinotti, Maria Gragnaniello, Salvatore Parlato, Jessica Centracchio, Emilio Andreozzi, Paolo Bifulco, Michele Riccio, Daniele Esposito

**Affiliations:** 1 Department of Electrical Engineering and Information Technologies, University of Naples Federico II, Via Claudio, 21, 80125 Naples, Italy; eliana.cinotti@unina.it (E.C.); maria.gragnaniello@unina.it (M.G.); salvatore.parlato@unina.it (S.P.); jessica.centracchio@unina.it (J.C.); emilio.andreozzi@unina.it (E.A.); paolo.bifulco@unina.it (P.B.); michele.riccio@unina.it (M.R.); 2 Department of Information and Electrical Engineering and Applied Mathematics, University of Salerno, Via Giovanni Paolo II, 132, 84084 Fisciano, Italy

**Keywords:** atrial fibrillation, edge AI, heart rate, wearables, neural networks, low-power, microcontroller, diagnosis

## Abstract

Atrial fibrillation (AF) is the most common type of heart rhythm disorder worldwide. Early recognition of brief episodes of atrial fibrillation can provide important diagnostic information and lead to prompt treatment. AF is mainly characterized by an irregular heartbeat. Today, many personal devices such as smartphones, smartwatches, smart rings, or small wearable medical devices can detect heart rhythm. Sensors can acquire different types of heart-related signals and extract the sequence of inter-beat intervals, i.e., the instantaneous heart rate. Various algorithms, some of which are very complex and require significant computational resources, are used to recognize AF based on inter-beat intervals (RR). This study aims to verify the possibility of using neural networks algorithms directly on a microcontroller connected to sensors for AF detection. Sequences of 25, 50, and 100 RR were extracted from a public database of electrocardiographic signals with annotated episodes of atrial fibrillation. A custom 1D convolutional neural network (1D-CNN) was designed and then validated via a 5-fold subject-wise split cross-validation scheme. In each fold, the model was tested on a set of 3 randomly selected subjects, which had not previously been used for training, to ensure a subject-independent evaluation of model performance. Across all folds, all models achieved high and stable performance, with test accuracies of 0.963 ± 0.031, 0.976 ± 0.022, and 0.980 ± 0.023, respectively, for models using 25 RR, 50 RR, and 100 RR sequences. Precision, recall, F1-score, and AUC-ROC exhibited similarly high performance, confirming robust generalization across unseen subjects. Performance systematically improved with longer RR windows, indicating that richer temporal context enhances discrimination of AF rhythm irregularities. A complete Edge AI prototype integrating a low-power ECG analog front-end, an ARM Cortex M7 microcontroller and an IoT transmitting module was utilized for realistic tests. Inferencing time, peak RAM usage, flash usage and current absorption were measured. The results obtained show the possibility of using neural network algorithms directly on microcontrollers for real-time AF recognition with very low power consumption. The prototype is also capable of sending the suspicious ECG trace to the cloud for final validation by a physician. The proposed methodology can be used for personal screening not only with ECG signals but with any other signal that reproduces the sequence of heartbeats (e.g., photoplethysmographic, pulse oximetric, pressure, accelerometric, etc.).

## 1. Introduction

Atrial fibrillation (AF) is the most common type of heart rhythm disorder worldwide, whose prevalence is constantly increasing [1,2,3,4,5,6]. AF is due to atrial abnormal electrical conductions and results in atria mechanical quivering rather than synchronous contraction. AF leads to an irregular heart rate that, in turn, significantly increase the risk of stroke, congestive heart failure, hypertension, diabetes, etc. [7]. The management of AF and of its severe complications generates an enormous economic burden on public health and healthcare services [8,9]. Therefore, early AF detection is of great importance [10]. However, AF episodes recognition remains challenging because of their paroxysmal nature (AF episodes can be very brief) and because episodes are generally asymptomatic and go unnoticed by patients [11].

International guidelines for AF screening recommend opportunistic and systematic patient screening [12]. Traditional 12-lead ECG or Holter recordings are very often unable to detect paroxysmal AF. In general, the longer the monitoring, the greater the likelihood of detecting AF episodes, and guidelines suggest very prolonged time patient monitoring [13]. However, prolonged ambulatory ECGs are expensive and require interpretation by healthcare professionals while implanted loop recorders require surgery and are not appropriate for population screening [14].

The enormous advantages of early detection of atrial fibrillation and the widespread use of smartphones have led to the development and commercialization of many personal devices that allow heart rhythm monitoring [15]. Since the main symptom of atrial fibrillation is irregular heart rhythm, these devices can provide useful early warnings [16,17]. Smartwatch, smart ring, fingertip on smartphone, smartphone inertial sensors, fitness band, handheld ECG, patch ECG, lead-based ECG, wrist worn devices are some examples of such personal devices [18,19]. FDA clearance gained by some commercial smartwatches to detect AF demonstrates remarkable interest in these technologies. Mainly, electrocardiogram or photoplethysmography signals are recorded by these devices and then processed to extract instantaneous heart rhythm and other information [15,20,21,22,23]. Other opportunistic strategies for recognizing arrhythmia have been to equip sphygmomanometers for blood pressure measurements or pulse oximetry devices with heart rhythm recognition capabilities, allowing for AF screening during routine operations [24].

In any case, whether it is electrocardiographic, photoplethysmographic, pulse oximetry, blood pressure, seismocardiographic, gyrocardiographic, forcecardiographic, or sphygmic wave recordings, it is essential to recognize the instantaneous heart rhythm and check for irregularities [25,26,27,28,29,30,31,32,33,34].

In addition to various deterministic algorithms, such as [35,36,37,38,39], many algorithms based on artificial intelligence have been proposed for the recognition of AF [40,41,42]. The use of increasingly complex algorithms generally requires considerable computing power, which is why many atrial fibrillation recognition software programs require smartphones or cloud platforms. This study proposes embedding artificial intelligence algorithms for AF recognition into common microcontrollers already used in portable or wearable devices. AF is detected exclusively on the basis of inter-beat intervals and can therefore be used by all types of sensors, whether they use electrocardiographic signals, photoplethysmography, or any other signals able to detect heartbeats. This makes this type of methodology applicable to a wide range of heart rate sensors.

This study analyzes sequences of inter-beat intervals of varying lengths to evaluate the performance of neural networks as the observation interval changes. In addition, a real-world implementation of the proposed methodology is presented. A long-term ECG recording system is interfaced with a microcontroller on which the neural network for AF recognition is implemented. When the heart rhythm analysis reveals a possible episode of AF, the ECG signal is sent to the cloud to allow the physician to validate the recognition.

## 2. Materials and Methods

### 2.1. Dataset Building

The MIT-BIH Atrial Fibrillation Database from PhysioNet [43] served as the data source. Of the 25 long-term ECG recordings available, each corresponding to a unique subject, 23 were included in the analysis. Recordings 00735 and 03665 were excluded because they contained only rhythm annotations and lacked accompanying ECG signal data.

Expert clinical annotations were used to extract the relevant signal segments. Only sinus rhythm and atrial fibrillation intervals were retained, while all other arrhythmias were removed during preprocessing in MATLAB^®^ R2023b. This resulted in a dataset comprising 113 h of sinus rhythm and 91 h of atrial fibrillation.

For each subject, inter-beat (RR) intervals (in seconds) were computed separately for sinus rhythm and atrial fibrillation. To ensure data reliability, intervals outside the range [0.25, 2] seconds (corresponding to 30–240 bpm) were removed, as these typically represent transitions between non-contiguous rhythm segments.

**Figure 1:**
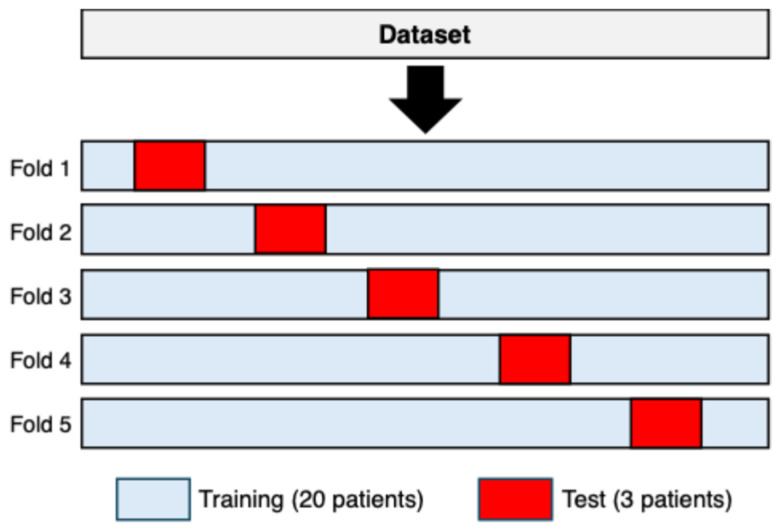
A 5-fold subject-wise split cross-validation used to construct the training and test sets. In each fold, three subjects (shown in red) were assigned exclusively to the test set, while the remaining twenty subjects (shown in light blue) were used for training. The composition of the test set changed across folds, ensuring that each subject appeared exactly once in a test partition and never in more than one fold.

From the cleaned data, sequences of 25 consecutive RR intervals were extracted for both rhythms. To achieve subject-independent validation, five distinct training–test configurations were generated. In each configuration, three subjects were assigned to the test set, while the remaining twenty subjects were used for training. The three test subjects varied across the five configurations so that no subject appeared in more than one test fold. This procedure yielded a 5-fold subject-wise split cross-validation with non-overlapping subject partitions [44], as illustrated in Figure 1.

In all datasets, each raw consisted of a sequence of 25 RR intervals (features) and a binary label (0 = sinus rhythm, 1 = atrial fibrillation). To ensure class balance, sinus rhythm samples were randomly removed to match the number of atrial fibrillation samples.

The entire procedure was repeated for sequence lengths of 50 and 100 RR intervals, producing five training–test dataset pairs for each sequence length.

**Figure 2:**
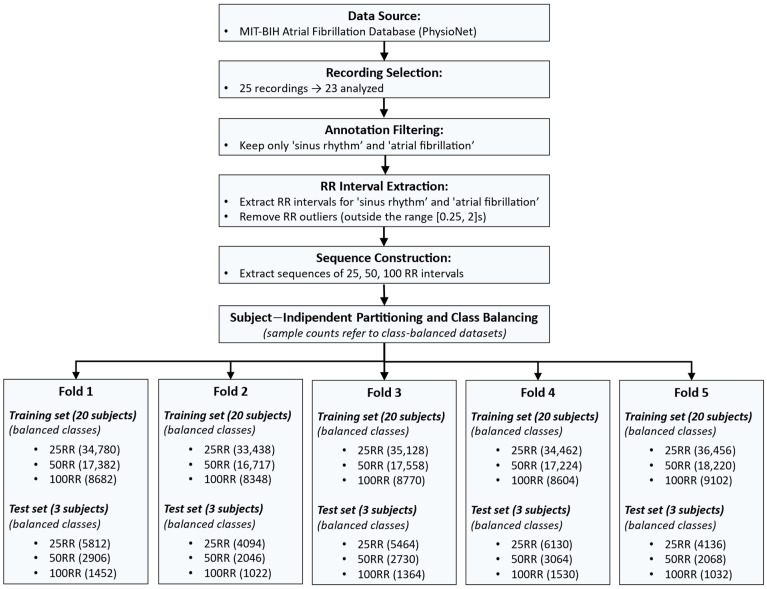
Workflow describing the dataset construction process. The pipeline includes: data selection from the MIT-BIH Atrial Fibrillation Database; annotation filtering to retain only sinus rhythm (SR) and atrial fibrillation (AF); RR interval extraction and outlier removal; sequence construction using 25, 50, and 100 RR intervals; subject-independent partitioning into five training–test folds (20 subjects for training and 3 for testing in each fold); and class balancing to equalize the number of SR and AF samples. The bottom section reports the number of samples (equally distributed in SR and AF) obtained in each fold for all sequence lengths.

All datasets were exported in Comma-Separated Values (CSV) format for subsequent use in the Edge Impulse web-based environment [45]. Figure 2 shows a flow chart of the procedure used to create the training and test sets for each fold.

### 2.2. Design of a Neural Network in Edge Impulse Environment

Using the “Edge Impulse” web application [45], for each fold the Training dataset with an 80/20 split was employed for model training and validation, respectively. Test dataset was reserved exclusively for testing on unseen data. The resulting Training-Validation/Test split was about 86%/14%.

A neural network was developed with the objective of enabling real-time atrial fibrillation detection on resource-constrained devices. The target deployment platform was a Cortex-M7 microcontroller, equipped with a 216 MHz clock speed, 340 KB of RAM, and 1 MB of flash memory.

**Figure 3:**
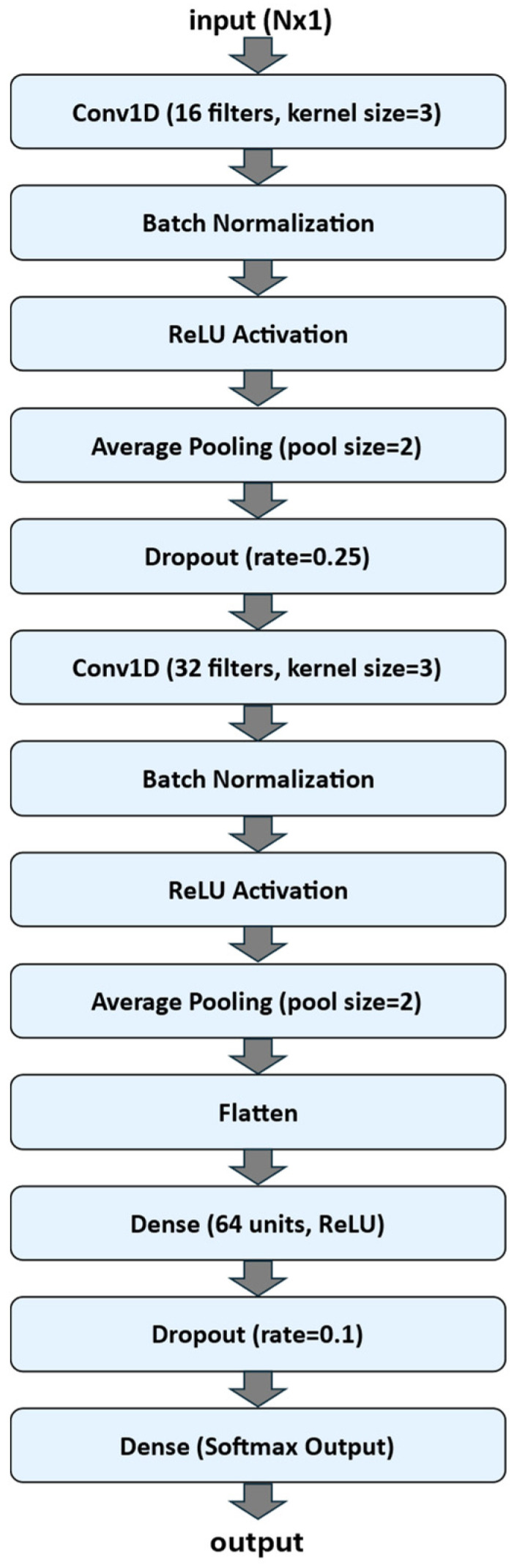
Flow chart of the 1D-CNN Neural Network Architecture.

A one-dimensional convolutional neural network (1D-CNN) was implemented using the TensorFlow Keras API to classify one-dimensional sequential data. The model was designed as a sequential architecture composed of convolutional, normalization, pooling, and fully connected layers. Figure 3 shows a flow chart of the 1D-CNN Neural Network Architecture.

The input to the network consisted of sequences of length N (N is equal to 25, 50 or 100, according to the dataset experimented) with a single feature channel (input size: N × 1). The first convolutional block included a Conv1D layer with 16 filters and a kernel size of 3, followed by Batch Normalization, a Rectified Linear Unit (ReLU) activation function, Average Pooling with a pool size of 2, and a Dropout layer (rate = 0.25) to reduce overfitting. The second convolutional block comprised 32 filters (kernel size = 3), again followed by Batch Normalization, ReLU activation, and Average Pooling (pool size = 2).

The output of the convolutional layers was flattened and passed to a fully connected (Dense) layer with 64 neurons and ReLU activation, followed by a Dropout layer (rate = 0.1). The final output layer employed a Softmax activation with a number of units corresponding to the number of target classes, enabling multi-class classification.

The model was trained using the Adam optimizer (learning rate = 0.0009, β_1_ = 0.9, β_2_ = 0.999) with categorical cross-entropy as the loss function and accuracy as the performance metric. Training was performed for up to 100 epochs with a batch size of 32. An Early Stopping callback monitored the validation loss and stopped training if no improvement was observed for 5 consecutive epochs, restoring the best-performing weights.

Computation was carried out on cloud-hosted CPU resources. The model was configured with both float32 and INT8 profiling.

### 2.3. Hardware and Software Architecture for Real-Time Testing

The proposed system integrates hardware and software components for real-time ECG acquisition, processing, atrial fibrillation detection and cloud-based data transmission.

#### 2.3.1. Hardware Setup for Simulating Realistic ECG Signals

A real-time validation was conducted to verify both the functional and temporal performance of the prototype under realistic operating conditions. The objective was to emulate physiological ECG signals, assess signal integrity, and confirm that acquisition, processing, and data transmission processes complied with real-time constraints.

To this end, as in previous study [39], a patient simulator circuit was designed to generate analog ECG signals replicating those of the subjects included in the Test dataset of fold 1 (see Section 2.1). The ECG recordings were preprocessed and subsequently stored on an SD card for playback during the real-time validation phase.

The simulation setup comprises a NUCLEO-F401RE (STMicroelectronics, Coppell, TX, USA) development board that sequentially reads ECG samples from the SD card at the original sampling frequency and transfers them to a MCP4725 12-bit DAC via an I^2^C interface. The DAC converts the digital samples into an analog signal ranging from 0 V to 3.3 V, with a resolution of 0.805 mV per step.

The analog signal was subsequently passed through a low-pass filter (cutoff frequency = 120 Hz) to remove quantization noise and attenuate high-frequency components. A 1:1000 voltage divider was then used to adjust the ECG amplitude to levels comparable to real ECG recordings (in the millivolt range). The conditioned signal was then fed into the MAX30003 front-end (Analog Devices, Wilmington, MA, USA), allowing the prototype to process it as if it were generated by real electrodes attached to a patient.

**Figure 4:**
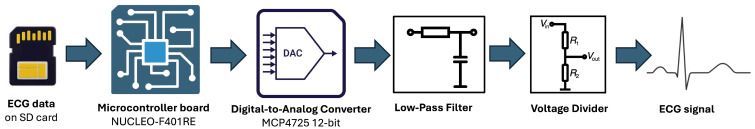
Hardware Architecture setup for generating realistic ECG signals.

The hardware architecture setup for generating realistic ECG signals is showed in Figure 4.

#### 2.3.2. Hardware Setup for Real-Time AF Detection and Data Transmission

**Figure 5:**
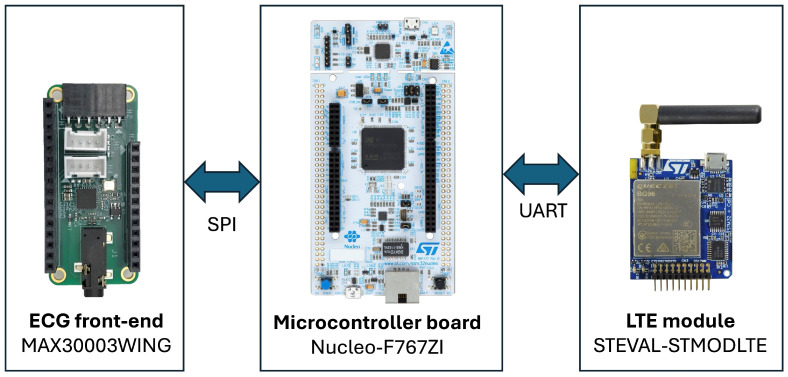
Hardware Architecture for real-time AF detection and data transmission.

The prototype is composed of three main modules (see Figure 5):

- *ECG front-end*: MAX30003WING (Analog Devices, Wilmington, MA, USA), based on the MAX30003 analog front-end for ECG monitoring. This chip integrates both analog and digital processing, including programmable gain, filtering options, and a built-in Pan–Tompkins QRS detection algorithm. It provides RR inter-beat interval estimation with minimal power consumption (~85 μW at 1.1 V).
- *Control unit*: Nucleo-F767ZI (STMicroelectronics, Coppell, TX, USA), featuring an STM32F767ZIT6 microcontroller with a 32-bit ARM Cortex-M7 core (up to 216 MHz). It receives data from the ECG front-end via SPI and transmits them to the communication module through UART.
- *Wireless communication unit*: STEVAL-STMODLTE (STMicroelectronics, Coppell, TX, USA), integrating a Quectel BG96 LTE Cat M1/NB1/EGPRS modem. This module provides low-power cellular connectivity for data upload to the cloud through Narrow Band-IoT protocols.

#### 2.3.3. Software Architecture for Real-Time AF Detection and Data Transmission

The software component of the proposed system was designed to operate efficiently within the constraints of an embedded environment, providing real-time data processing, decision-making, and wireless communication. The firmware was developed using Mbed OS [46], an open-source real-time operating system optimized for ARM microcontrollers, which offers multitasking capabilities, hardware abstraction, and integrated libraries for communication protocols and peripheral management.

All decision-making processes are managed exclusively through the proposed 1D-CNN (see Section 2.2), thus eliminating the need for traditional analytical algorithms such as Lorenz-based classifiers [39].

**Figure 6:**
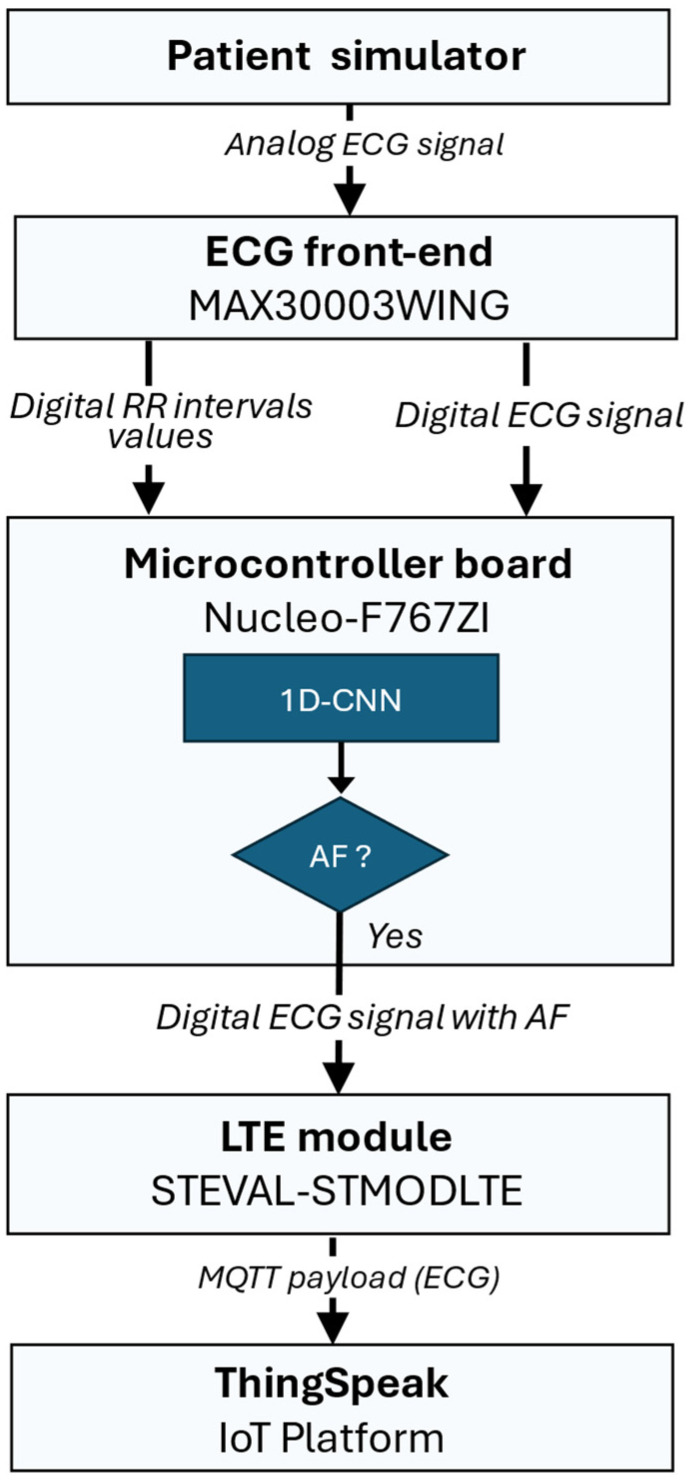
Signal flow of the embedded system for real-time AF detection and data transmission: the ECG signal is acquired by the MAX30003 front-end, which computes RR intervals and sends them to the Nucleo-F767ZI microcontroller for real-time neural inference. If an AF is detected, the STEVAL-STMODLTE LTE module publishes the corresponding ECG window to the ThingSpeak IoT platform for physician validation.

The signal flow of the embedded system for real-time AF detection and data transmission is illustrated in Figure 6 and is structured into the following main stages:

- *Data Acquisition and Buffering:* ECG signals are continuously sampled at 125 Hz with 18-bit resolution using the MAX30003 front-end (internally operating at 32 KHz), with an ECG gain of about 160 V/V and a bandwidth of 0.5–40 Hz. Both raw ECG samples and computed RR intervals are stored in independent memory buffers.
- *Neural Inference:* when the RR buffer reaches the predefined window size (e.g., 25, 50, or 100 samples), the firmware preprocesses the data and invokes the Edge Impulse inference engine compiled directly into the microcontroller firmware. The model outputs the result of the inference.
- *Connectivity and Data Publication*: if a sequence of RR intervals is classified as an AF event, the system transmits the corresponding ECG window to ThingSpeak cloud service [47] for physician validation. In details, a cellular connection is established through the LTE module (STEVAL-STMODLTE) using the Mbed OS Cellular stack and the MQTT protocol is used to publish a JSON-formatted payload containing the ECG window. When a normal rhythm is detected, the data buffers are cleared, and a new acquisition cycle begins.

### 2.4. Real-Time Performances

The analyses presented in this section refer exclusively to the quantized (INT8) 1D-CNN model trained on sequences of 100 RR intervals, as this configuration achieved the best performance in terms of classification accuracy among the INT8 tested models (see Section 3.2).

#### 2.4.1. Temporal Measurements

To verify the real-time performance of the proposed system, a set of timing tests was conducted to evaluate the execution time of the main firmware routines, including ECG data acquisition, neural inference, and data publication.

The analysis was performed using a logic state analyzer connected to the NUCLEO-F767ZI development board. The digital pin D14 was configured as a synchronization signal: the firmware set the pin to a high logic level at the beginning and to a low level at the end of the section under test. This method allowed precise measurement of the execution time and periodicity of each task.

The measurements focused on the timing of ECG interrupt events, RR interval detection, neural inference triggered by buffer completion, and MQTT-based data transmission.

#### 2.4.2. Power Consumption Measurements

To characterize the prototype power consumption under different operating modes, current measurements were carried out using the Power Profiler Kit II (PPK2, Nordic Semiconductor, Trondheim, Norway) [48].

The tests were performed separately for the main hardware subsystems:

- the MAX30003 ECG front-end;
- the NUCLEO-F767ZI microcontroller board;
- the STEVAL-STMODLTE LTE communication module.

For the NUCLEO board, the PPK2 was connected through the dedicated IDD jumpers to monitor current consumption during different operational phases, including interrupt handling, AI inference, and data publication.

The ECG front-end and LTE module were powered individually during the tests to allow independent profiling of their respective energy consumption.

The analysis aimed to determine both the average and peak currents associated with (i) ECG interrupt events, (ii) RR interval processing, (iii) neural inference, (iv) data publication, and (v) idle intervals between two transmission events. All experiments were performed under identical supply and environmental conditions to ensure reproducibility.

## 3. Results

### 3.1. Performance of the Quantized (INT8) and Float32 1D-CNN Models Across the Five Validation Folds

This section reports the performance of the 1D-CNN models, both quantized (INT8) and float32, evaluated using the 5-fold subject-wise split cross-validation scheme.

The evaluation was performed separately for the three RR-interval configurations (25 RR, 50 RR, 100 RR), using the validation datasets associated with each fold. Model performance was assessed using accuracy, precision, recall, F1-score, AUC-ROC, and validation loss. For each configuration, the final performance is expressed as the mean ± standard deviation computed across the five folds.

Table 1 summarizes the aggregated metrics for the INT8 and float32 models across the three RR-interval lengths.

Across all RR-interval configurations, both the quantized INT8 and float32 models achieved high performance across the five validation folds. For the 25 RR datasets, the INT8 model achieved a mean validation accuracy of 0.979 ± 0.003, with precision, recall, and F1-score equally balanced. The AUC-ROC (0.979 ± 0.003) indicates a strong ability to discriminate between sinus rhythm and atrial fibrillation. The float32 model exhibited nearly identical performance, with slightly lower validation loss. Performance further improved with longer RR-interval sequences. The 50 RR and 100 RR datasets showed accuracy values above 0.986 and 0.989, respectively, with extremely small variability across folds (standard deviation ≈ 0.003). These results confirm that increasing the number of consecutive RR intervals provides richer temporal information, improving classification robustness.

Overall, the quantized INT8 model matched the float32 baseline in all metrics, thus demonstrating that memory-efficient deployment on low-power edge devices can be achieved without loss of performance.

To complement these results, Table 2 reports the memory footprint measured on the target microcontroller for all trained models. Memory requirements were determined exclusively by the RR-interval window length and the numerical precision (INT8 vs. float32) and were consistent across the five cross-validation folds. For the quantized INT8 versions, peak RAM usage ranged from 6.1 KB (25 RR) to 8.5 KB (100 RR), while flash usage increased from 48.6 KB to 86.6 KB. The float32 counterparts required substantially larger memory resources, with RAM usage between 6.6 KB and 16.0 KB and flash consumption ranging from 69.9 KB to 221.9 KB.

These results confirm that INT8 quantization reduces flash memory requirements by up to a factor of three, while also achieving lower RAM usage.

Representative learning curves for the three RR datasets (fold 1), further illustrate the INT8 model training behavior.

**Figure 7:**
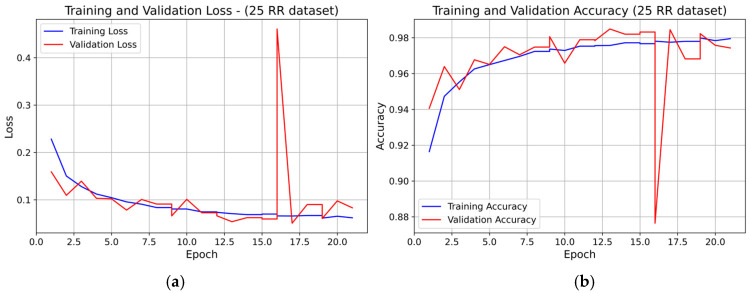
Learning curve of the 1D-CNN (INT8) model on the 25 RR dataset (fold 1): panel (**a**) Training and validation loss; panel (**b**) Training and validation accuracy.

For the 25 RR dataset, both training and validation loss decreased overall during the first 14–15 epochs, with small oscillations (see Figure 7). A marked transient spike in validation loss is visible at epoch 16, after which the curve returns to stable values. The validation accuracy closely followed the training accuracy for the entire training process, except for a brief drop corresponding to the loss spike, and no systematic divergence was observed. Overall, the model showed stable learning dynamics with no evidence of persistent overfitting.

**Figure 8:**
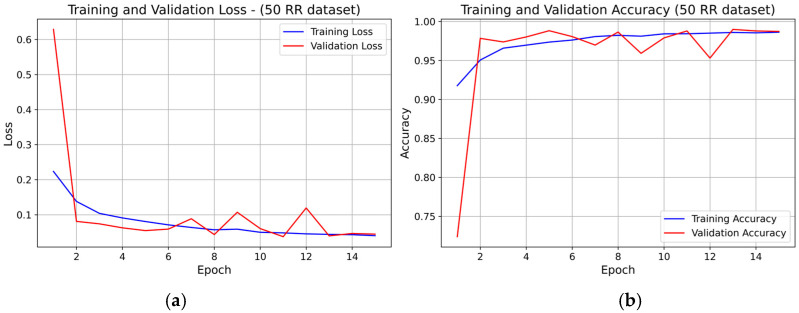
Learning curve of the 1D-CNN (INT8) model on the 50 RR dataset (fold 1): panel (**a**) Training and validation loss; panel (**b**) Training and validation accuracy.

For the 50 RR dataset, the training and validation loss curves (see Figure 8) showed rapid convergence within the first 4–5 epochs, followed by stable behavior with minor fluctuations. Validation accuracy increased sharply in the early epochs and then remained stable, closely tracking the training accuracy The small rise in validation loss toward the final epochs did not lead to accuracy degradation, confirming stable convergence and absence of overfitting.

**Figure 9:**
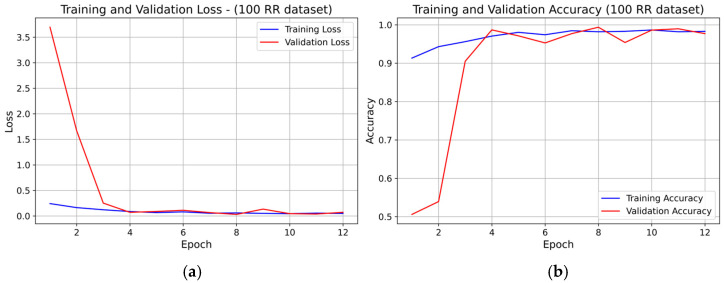
Learning curve of the 1D-CNN (INT8) model on the 100 RR dataset (fold 1): panel (**a**) Training and validation loss; panel (**b**) Training and validation accuracy.

For the 100 RR dataset, the learning curves (see Figure 9) also exhibited rapid convergence, with both training and validation loss stabilizing around epoch 5–6. Early stopping was likely triggered near epoch 8, consistent with the minimal validation loss region. Training and validation accuracy overlapped for nearly all epochs, indicating very good generalization and no overfitting.

Across all three RR-interval configurations, no evidence of systematic overfitting was observed. Validation accuracy consistently followed training accuracy, and the loss curves remained stable after convergence. The slight fluctuations in validation loss did not affect long-term convergence. Increasing the RR-interval window (from 25 RR to 100 RR) resulted in smoother curves, faster convergence, and more stable learning behavior.

### 3.2. Performance of the Quantized (INT8) and float32 1D-CNN Models Across the Five Test Folds

This section reports the performance of the 1D-CNN models, quantized (INT8) and float32, evaluated on the five independent test sets generated through the 5-fold subject-wise split cross-validation scheme. Each test set contains sequences extracted from three subjects not included in the corresponding training fold.

Performance was evaluated using accuracy, precision, recall, F1-score, AUC-ROC, and test loss. For each RR-interval configuration (25 RR, 50 RR, and 100 RR), the results are expressed as the mean ± standard deviation computed across the five test folds. This provides a robust estimate of model performance, where inter-subject variability can significantly influence classification results.

Table 3 summarizes the aggregated performance metrics for both INT8 and float32 models.

Across all test folds, both INT8 and float32 1D-CNN models demonstrate robust and consistent classification performance, with all metrics showing minimal variability across subjects. A clear trend emerges as the RR-interval window increases: using longer temporal sequences systematically improves all performance indicators.

For the 25 RR dataset, the INT8 model achieves an accuracy of 0.963 ± 0.031, closely matching the float32 performance (0.962 ± 0.031). Precision, recall, and F1-score follow the same pattern. The most noticeable difference between INT8 and float32 appears in the test loss (0.178 vs. 0.118), which proves to be more sensitive to numerical quantization, even when classification metrics remain aligned.

For the 50 RR dataset, classification performance increases across all metrics (accuracy: 0.976 ± 0.022 for INT8 and 0.975 ± 0.022 for float32). Precision, recall, F1-score and AUC-ROC remain tightly clustered around identical values for the two models, with differences well below 0.001. The INT8 test loss (0.108 ± 0.078) remains slightly higher than the float32 baseline (0.077 ± 0.055), but this gap does not translate into meaningful differences in predictive behavior.

In the 100 RR configuration, the INT8 and float32 models exhibit their highest performance, achieving accuracies of 0.980 ± 0.023 and 0.980 ± 0.021, respectively, together with equally high values in precision, recall, F1-score, and AUC-ROC. Moreover, although the INT8 model reports a higher test loss (0.177 vs. 0.081), its classification performance is fully preserved and closely matches the float32 implementation.

Overall, model performance improves by approximately +1.3% when moving from 25 RR to 50 RR, and by an additional +0.4% when extending to 100 RR, confirming that longer RR windows provide richer temporal context and yield more reliable discrimination between AF and sinus rhythm. Significantly, INT8 and float32 models exhibit nearly identical behavior across all metrics, with differences typically below 0.001. This shows that quantization does not degrade AF classification accuracy and supports the deployment of the INT8 model on low-power embedded platforms, where memory footprint and computational load are substantially reduced without compromising diagnostic performance.

To further investigate the classification behavior of the models, confusion matrices were computed by aggregating the test predictions across all five folds, thus producing a global confusion matrix for each RR-interval configuration. Since each test fold contains samples from mutually exclusive subjects, summing the true positives, true negatives, false positives, and false negatives across folds yields a valid and meaningful evaluation of the model performance over the entire cross-validation procedure [49,50].

**Figure 10:**
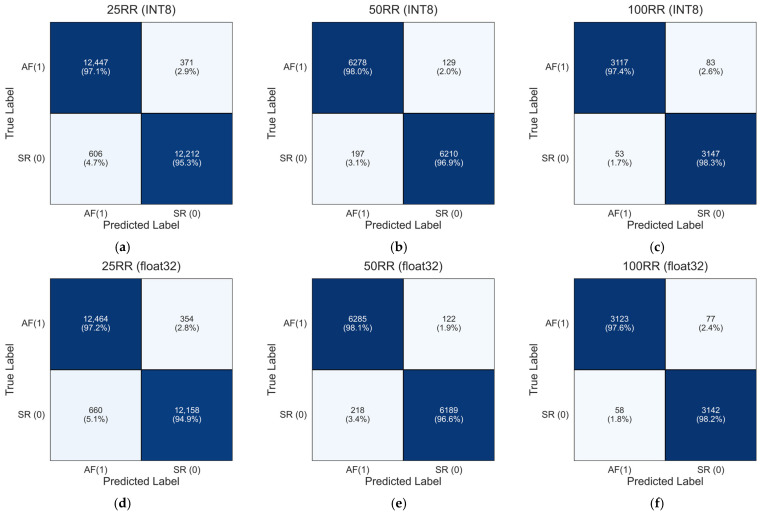
Aggregated confusion matrices for the INT8 and float32 1D-CNN model across the five subject-independent test folds. The matrices were obtained by summing the confusion matrices of each fold, providing a global assessment of the model ability to discriminate between sinus rhythm (SR) and atrial fibrillation (AF) for the 25 RR, 50 RR, and 100 RR configurations: (**a**) 25RR INT8; (**b**) 50RR INT8; (**c**) 100RR INT8; (**d**) 25RR float32; (**e**) 50RR float32; (**f**) 100RR float32.

Figure 10 reports the resulting aggregated confusion matrices for the INT8 and float32 models for each dataset of 25 RR, 50 RR and 100 RR, computed as in Equation (1):

(1) $${CM}_{j}=\sum_{k=1}^{5}{CM}^{\left(k\right)};\;j\in \left\{25\;RR,50\;RR,100\;RR\right\},\;k=fold$$

The aggregated confusion matrices in Figure 10a–f align closely with the mean performance metrics reported in Table 3. For the 25 RR dataset, the INT8 model (panel a) achieves global true positive and true negative rates of 97.1% and 95.3%, respectively, whereas the float32 model (panel d) shows nearly identical values (97.2% and 94.9%). For the 50 RR configuration, performance improves for both models. The INT8 version (panel b) reaches 98.0% true positives and 96.9% true negatives, while the float32 model (panel e) achieves 98.1% and 96.6%, respectively. The reduction in misclassification with increasing RR-interval length demonstrates the benefit of incorporating longer temporal patterns. For the 100 RR dataset, both models achieve their highest performance levels. The INT8 model (panel c) attains 97.4% (AF) and 98.3% (SR), while the float32 model (panel f) achieves 97.6% and 98.2%, respectively.

Finally, the aggregated confusion matrices reinforce the robustness of both INT8 and float32 models across the five test folds. The extremely small differences between the two implementations confirm that the INT8 quantization does not degrade classification performance, consistent with the near-identical values observed in Table 3.

### 3.3. Results of Real-Time Testing

#### 3.3.1. Temporal Performance

**Figure 11:**
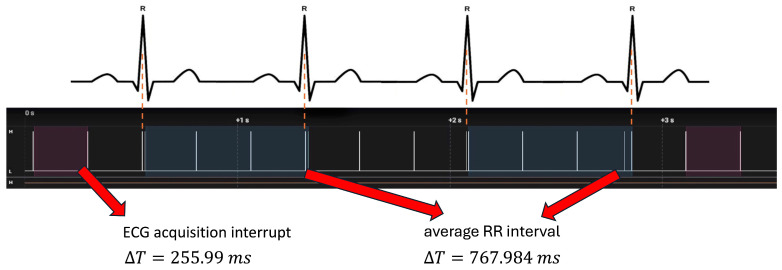
Temporal performance test using a logic state analyzer. Upper panel: ECG signal with highlighted R-peaks. Lower panel: timing of interrupt events associated with ECG acquisition. The acquisition interrupt occurs every ΔT = 255.99 ms (highlighted in dark wine), while the average RR interval is ΔT = 767.984 ms (highlighted in dark slate blue), consistent with a physiological heart rate of approximately 78 bpm.

As shown in Figure 11, the ECG acquisition interrupt occurred every 255.99 ms, which corresponds to the expected period of the FIFO buffer (32 samples × 8 ms = 256 ms). The corresponding processing block lasted 2.49 ms. The average RR interval was approximately 768 ms, consistent with a physiological heart rate of about 78 bpm and in agreement with the ECG signal acquired in parallel.

Neural inference was triggered when 100 RR intervals were accumulated. The total inference block (including data management) required 23.16 ms, whereas the AI computation alone lasted approximately 2.7 ms. The time between two inference events was 78.27 s, consistent with the acquisition of 100 RR inter-beat intervals at the given sampling frequency.

Following the detection of an atrial fibrillation (AF) episode, the system-initiated data publication via MQTT. The transmission phase lasted 2.58 s, representing the time required to serialize and transmit the ECG vector to the cloud.

#### 3.3.2. Power Consumption Analysis

**Figure 12:**
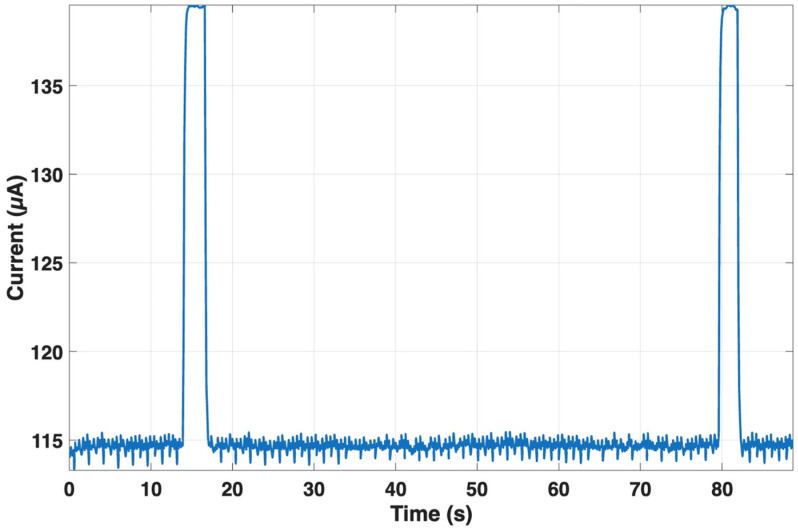
Current consumption of the MAX30003 front-end during continuous RR monitoring. The current remains stable at ~115 µA, with brief peaks up to ~140 µA during interrupt generation.

For the MAX30003 front-end, the current remained nearly constant at 115 µA, with peaks up to about 140 µA during interrupt generation, confirming its negligible contribution to total energy consumption (see Figure 12). The stability of the power profile demonstrates that the ECG front-end introduces a minimal load on the overall system budget.

**Figure 13:**
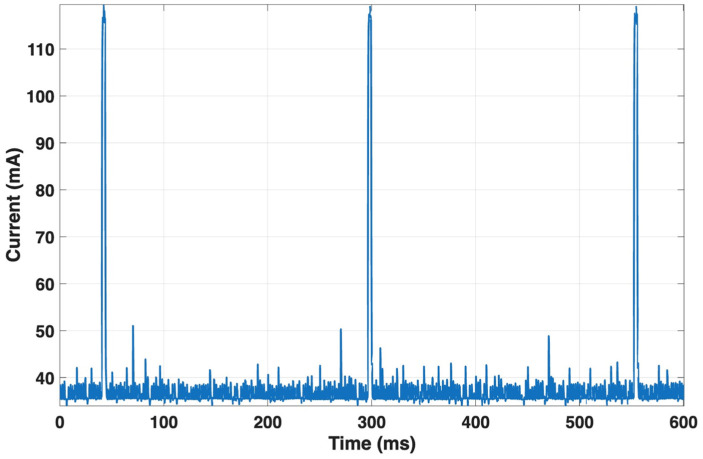
Current consumption of the NUCLEO-F767ZI during acquisition interrupts. The board draws an average of ~96.7 mA with peaks up to ~120 mA during the interrupt routine.

For the NUCLEO-F767ZI, the average current during acquisition interrupts was about 96.7 mA, with a peak value of approximately 120 mA (see Figure 13), consistent with an event duration of about 256 ms.

The current profile between consecutive R-peaks showed similar values (mean = 96.7 mA; peak = 120.1 mA) for an RR interval of about 0.75 s. Neural inference lasted only 2 ms, with a mean current of 113.6 mA and a peak of 119.7 mA. The MQTT publication phase, lasting 2.43 s, exhibited a mean current of 57.4 mA and a peak of 111.3 mA, reflecting the activation of the communication stack. Between two consecutive publications (about 56.4 s apart), the current averaged 95.3 mA. Table 4 summarizes the main results relating to the microcontroller power consumption during the various operations experimented.

**Figure 14:**
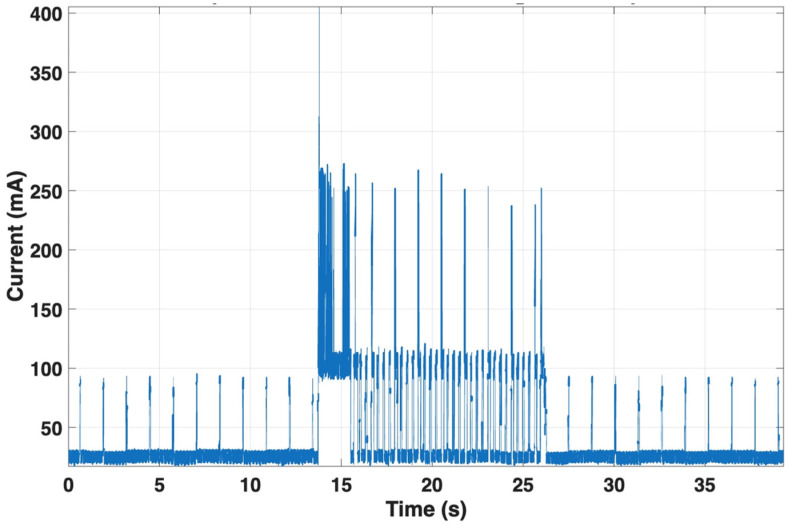
Current consumption of the LTE module during MQTT transmission. During the uplink event, the module draws an average of ~61.1 mA with peaks up to ~405.4 mA over 12.6 s. The burst-like current pattern characteristic of cellular transmission makes this phase the main contributor to overall energy consumption.

Finally, the LTE module exhibited the highest energy demand. During MQTT transmission, current averaged 61.1 mA, with peaks reaching 405.4 mA over a 12.6 s event (see Figure 14). The profile showed characteristic burst behavior associated with cellular uplink transmission. This phase dominates the device overall power consumption.

## 4. Discussion and Conclusions

The use of a one-dimensional convolutional neural network (1D-CNN) is particularly well suited for the classification of cardiac rhythms such as Atrial Fibrillation (AF) and Sinus Rhythm (SR) when the input data consist of sequences of inter-beat (RR) intervals [51,52]. In AF, these intervals are characterized by high irregularity and the absence of consistent periodicity, whereas SR exhibits relatively stable and predictable temporal patterns.

A 1D-CNN can efficiently learn these local temporal dependencies and morphological patterns within a short window of RR intervals (e.g., 25, 50, or 100 consecutive beats). The convolutional filters act as feature detectors that automatically extract rhythm-specific signatures, such as sudden variations, repeating patterns, or irregular fluctuations. The subsequent pooling operations provide translational invariance and reduce noise sensitivity, while fully connected layers integrate the extracted temporal features to form a global representation for rhythm classification.

Compared with traditional machine learning approaches based on engineered heart rate variability (HRV) metrics, this architecture offers the advantages of automatic feature learning, robustness to signal variability, and efficient training on compact 1D representations [53]. Consequently, the proposed CNN framework provides a powerful and computationally lightweight method for distinguishing AF from SR directly from short RR interval sequences, making it suitable for both real-time and large-scale arrhythmia screening applications.

Using a 5-fold subject-wise split cross-validation scheme, both the quantized INT8 and float32 models exhibited consistently high classification performance. Validation metrics (Table 1) showed near-identical accuracy, precision, recall, F1-score, and AUC-ROC values between INT8 and float32 models for all RR-window lengths. Performance improved systematically with longer sequences: accuracy increased from 0.979 (25 RR) to 0.986 (50 RR) and 0.989 (100 RR). This confirms that richer temporal context enhances the model ability to capture the complex temporal irregularities typical of AF.

Test performance across all five folds (Table 3) confirmed these findings. Accuracy ranged from 0.963 ± 0.031 (25 RR) to 0.980 ± 0.023 (100 RR) for the INT8 model, with the float32 model achieving essentially identical values (differences ≤ 0.001). The low standard deviations highlight the stability of the model despite inter-subject variability and demonstrate the reliability of the subject-independent evaluation. These results confirm that quantization does not degrade the discriminative capability of the model.

Memory profiling on the target microcontroller (Table 2) showed that resource usage depends exclusively on the model architecture, input dimension, and numerical precision, and remains identical across the five folds. For INT8 models, RAM usage ranged between 6.1–8.5 KB, while flash memory consumption ranged between 48.6–86.6 KB. In contrast, float32 models required substantially more memory, with RAM usage up to 16 KB and flash memory up to 221.9 KB, approximately three times larger than their INT8 counterparts. These results confirm that INT8 quantization drastically reduces memory requirements while maintaining performance, enabling efficient on-device inference on constrained hardware [54].

Real-time testing validated the system operational feasibility on embedded hardware, demonstrating deterministic timing, low-latency inference (23.16 ms total; 2.7 ms AI computation), and reliable data transmission (2.58 s MQTT publication) fully compliant with real-time constraints. Power analysis revealed high energy efficiency, with the ECG front-end drawing only about 115 µA and the LTE module identified as the primary energy consumer, although the network communication is triggered only upon AF detection.

Compared with our previous implementation [39] based on the Lorentz algorithm executed on the ThingSpeak cloud platform, the present study introduces a substantial advancement by embedding the AF detection algorithm directly within the microcontroller. In the earlier architecture, RR interval sequences were periodically transmitted to the cloud (every two minutes) for analysis, which required continuous connectivity and frequent data transfers. In the proposed system, AF recognition is entirely performed locally through the embedded 1D-CNN, thus eliminating the need for remote computation and enabling fully autonomous operation. This shift from cloud-based analytics to on-device intelligence not only simplifies system architecture but also reduces latency and enhances data privacy and reliability.

From a diagnostic standpoint, the improvement in classification performance is remarkable. Using the same MIT-BIH Atrial Fibrillation Database [43], the previous system achieved an accuracy of 0.88, whereas the present 1D-CNN model reached an accuracy of about 0.98 when trained and tested on sequences of 100 RR intervals. This substantial increase highlights the superior capability of the neural network to extract and interpret the complex temporal irregularities characteristic of atrial fibrillation directly from the heartbeat interval sequence.

Furthermore, the proposed edge AI architecture provides substantial advantages in terms of energy efficiency. As demonstrated, the LTE transmission module represents the main source of power consumption, and in the previous system the need for frequent RR-sequence uploads led to considerable energy expenditure. In the current implementation, by performing inference locally and activating data transmission only when a potential AF event is detected, overall power consumption is drastically reduced. This improvement significantly extends the operational lifetime of the wearable device and enhances its suitability for long-term, continuous monitoring applications.

In summary, the transition from a cloud-based Lorentz algorithm to an embedded 1D-CNN model has enabled a system that is not only more accurate but also more autonomous and energy-efficient. The integration of artificial intelligence directly at the edge demonstrates the feasibility of real-time, low-power AF detection on wearable platforms, paving the way for next-generation personal health monitoring systems that combine diagnostic accuracy with practical sustainability.

Finally, this study has some limitations. First, the behavior of the proposed 1D-CNN has not been evaluated on mixed sinus rhythm/atrial fibrillation segments or in the presence of other arrhythmias, where rhythm transitions and ectopic activity may introduce additional variability. Moreover, although the considered dataset includes approximately 200 h of ECG recordings, it reflects a relatively small cohort of only 23 subjects. Future work should therefore assess the generalizability of the system on a larger and more diverse population and extend the analysis to more complex rhythm patterns.

## Figures and Tables

**Table 1:** Performance metrics (mean ± standard deviation) of the 1D-CNN models evaluated on the five cross-validation folds for the 25 RR, 50 RR, and 100 RR validation datasets.

Dataset	Model	Accuracy	Precision	Recall	F1-score	AUC-ROC	Validation Loss
25 RR	INT8	0.979 ± 0.003	0.979 ± 0.003	0.979 ± 0.003	0.979 ± 0.003	0.979 ± 0.003	0.088 ± 0.011
	Float32	0.979 ± 0.004	0.979 ± 0.004	0.979 ± 0.004	0.979 ± 0.004	0.979 ± 0.004	0.069 ± 0.013
50 RR	INT8	0.986 ± 0.003	0.986 ± 0.003	0.986 ± 0.003	0.986 ± 0.003	0.986 ± 0.003	0.061 ± 0.026
	Float32	0.986 ± 0.003	0.986 ± 0.003	0.986 ± 0.003	0.986 ± 0.003	0.986 ± 0.003	0.047 ± 0.014
100 RR	INT8	0.989 ± 0.003	0.989 ± 0.003	0.989 ± 0.003	0.989 ± 0.003	0.989 ± 0.003	0.055 ± 0.016
	Float32	0.989 ± 0.003	0.989 ± 0.003	0.989 ± 0.003	0.989 ± 0.003	0.989 ± 0.003	0.040 ± 0.011

**Table 2:** Peak RAM usage and flash usage of the INT8 and float32 1D-CNN models for the 25 RR, 50 RR, and 100 RR datasets. Values refer to deployed models on the target microcontroller.

Dataset	Model	Peak RAM Usage [KB]	Flash Usage [KB]
25 RR	INT8	6.1	48.6
	Float32	6.6	69.9
50 RR	INT8	6.9	62.5
	Float32	9.7	125.8
100 RR	INT8	8.5	86.6
	Float32	16.0	221.9

**Table 3:** Performance metrics (mean ± standard deviation) of the 1D-CNN models evaluated on the five test folds for the 25 RR, 50 RR, and 100 RR datasets.

Dataset	Model	Accuracy	Precision	Recall	F1-score	AUC-ROC	Test Loss
25 RR	INT8	0.963 ± 0.031	0.964 ± 0.031	0.963 ± 0.031	0.963 ± 0.031	0.963 ± 0.031	0.178 ± 0.160
	Float32	0.962 ± 0.031	0.962 ± 0.031	0.962 ± 0.031	0.962 ± 0.031	0.962 ± 0.031	0.118 ± 0.091
50 RR	INT8	0.976 ± 0.022	0.976 ± 0.022	0.976 ± 0.022	0.976 ± 0.022	0.976 ± 0.022	0.108 ± 0.078
	Float32	0.975 ± 0.022	0.975 ± 0.022	0.975 ± 0.022	0.975 ± 0.022	0.975 ± 0.022	0.077 ± 0.055
100 RR	INT8	0.980 ± 0.023	0.980 ± 0.022	0.980 ± 0.023	0.980 ± 0.023	0.980 ± 0.023	0.177 ± 0.210
	Float32	0.980 ± 0.021	0.980 ± 0.020	0.980 ± 0.021	0.980 ± 0.021	0.980 ± 0.021	0.081 ± 0.088

**Table 4:** Summary of microcontroller power consumption during various operations.

Operation	Duration of Measurement	Average Current (mA)	Peak Current (mA)
Acquisition Interrupt	256 ms	96.7	119.7
RR Interval	752 ms	96.7	120.1
Inference	2 ms	113.6	119.7
MQTT Publication	2.43 s	57.4	111.3
Between Publications	56.4 s	95.3	120.7

## Data Availability

The original data presented in the study are openly available in [PhysioNet] at https://physionet.org/content/afdb/1.0.0/ (accessed on 8 August 2025).
